# Leg Autotomy and Regeneration in Crab Spiders *Ebrechtella tricuspidata* (Araneae: Thomisidae)

**DOI:** 10.3390/insects17080833

**Published:** 2026-08-11

**Authors:** Xueqing Li, Xuanda Chen, Zhen Gong, Yu Peng

**Affiliations:** 1 Arachnid Resource Centre of Hubei & Hubei Key Laboratory of Regional Development and Environmental Response, Faculty of Resources and Environmental Sciences, Hubei University, Wuhan 430062, China; xueqingli1212@163.com (X.L.); chenxunda2024@163.com (X.C.); 2 Xiangyang Plant Protection Station, Xiangyang 441021, China; gongzhen1900@163.com

**Keywords:** *Ebrechtella tricuspidata*, leg autotomy, regeneration, predation, mating

## Abstract

Arachnids evade predation by intentionally sacrificing body parts, with leg autotomy playing a crucial role in their survival and reproductive strategies. Our study primarily employed field surveys and laboratory experiments to examine the frequency of autotomy and its effects on predation and mating in *Ebrechtella tricuspidata* (Araneae: Thomisidae). Leg loss was common in wild populations, with approximately 17.11% of all surveyed spiders missing at least leg, the most frequently lost leg is one of the second pair of appendages, and the highest rate of autotomy occurs in July. Additionally, we found that some individuals exhibit regenerative capacity following autotomy. We focused on the effects of leg autotomy on *E. tricuspidata* in two contexts: predation and mating. In laboratory conditions, the loss of two legs significantly impacted the individual’s predatory efficiency. Additionally, individuals missing two legs exhibited significantly poorer mating performance than intact individuals. We suggest that *E. tricuspidata* may employ a synergistic strategy of “autotomy-regeneration” to mitigate survival risks, thereby reducing the negative impacts of leg loss on predation and mating.

## Simple Summary

Leg autotomy is a common self-defense behavior exhibited by spiders when they encounter danger. As an ambush hunter, *Ebrechtella tricuspidata* (Araneae: Thomisidae) is commonly found among flowers and grasses, and leg autotomy frequently occurs in the wild. However, it remains unclear how leg autotomy affects this species. In this study, we surveyed autotomy in natural populations of *E. tricuspidata*. *E. tricuspidata* exhibits a moderate level of leg autotomy, and individuals that had undergone autotomy were capable of regenerating their lost legs. We also tested whether autotomy affects an individual’s subsequent hunting and mating capacities. Under our laboratory conditions, we found that the loss of two legs had a significant impact on predation efficiency within 30 min. At the same time, we also found that individuals missing multiple legs exhibit significantly weaker mating performance than intact ones. Our findings suggest that *E. tricuspidata* may resist predator attacks through autotomy and offset its effects on predation and mating through regeneration.

## 1. Introduction

Animals have many ways to avoid being eaten, ranging from predator-avoidance behaviors such as visual camouflage, displaying threatening behaviors, or adopting aposematic coloration [1]. Of these “last gasp” antipredator behaviors, autotomy stands out as an important and extensively studied. Autotomy, the voluntary self-amputation of an appendage, is a phenomenon observed across multiple taxonomic groups, including reptiles [2] and echinoderms [3], and it is especially common among the arthropods, such as insects [4], centipedes [5], crustaceans [6], and arachnids [7].

The frequency of appendage loss varies significantly among different arthropod species. Field surveys of various spiders indicate that approximately 5% to 20% of individuals exhibit appendage loss [8]. And this rate further varies by species, age, sex, and season, sometimes reaching 30% to 40% [9]. Among wolf spiders in natural populations, approximately 8% to 32% of individuals lack at least one leg [10]. In the crab spider *Misumenoides formosipes*, the leg-loss rate in males can increase from 6.3% to 41.6% throughout the mating season [9], suggesting that autotomy confers an evolutionary advantage.

Autotomy proves highly effective for predators that capture prey by grasping specific body parts, responding to potentially fatal poisoning [11] or damaged legs [12], but it also entails high costs. Injured individuals often find themselves at a disadvantage when competing with intact counterparts, facing issues such as the necessity to expend additional energy to regenerate the lost body part, reduced foraging ability, lower reproductive success, diminished competitive capacity, decreased mating success, and increased predation risk [6]. Although some species can regrow lost appendages [13], this process requires substantial energy expenditure. Overall, for spiders investing resources in regeneration, the cost of losing legs may be particularly high. Thus, when animals face the choice between leg loss and death, the value of autotomy becomes evident.

Existing research on autotomy in arachnids has largely focused on wolf spiders, whilst quantitative and systematic studies on crab spiders remain insufficient. In particular, there is a lack of field and laboratory evidence regarding the effects of autotomy on appendage regeneration, predation efficiency, mating performance, and long-term adaptive survival, leaving a great gap in the relevant research. Crab spiders are non-orb-weaving and wandering predators [14]. Their first two pairs of legs are long and robust, serving primarily for locomotion, predation, and courtship [15], making them ideal subjects for studying the costs of leg autotomy. *Ebrechtella tricuspidata* is a common ambush predator found in agricultural ecosystems and urban parks [16], which has a wide distribution and is easy to rear [17]. However, no studies have been reported on the patterns of leg autotomy, regenerative capacity, or functional costs associated with this species.

Our study focuses on *E. tricuspidata*, using field population surveys and assessments of appendage regeneration to investigate the impact of autotomy on predation and mating performance. The aim is to fill a research gap on the ecological costs of leg autotomy in crab spiders and to provide a foundation for agricultural ecology and behavioral ecology. We would like to examine the following hypotheses: (1) autotomy is a widespread phenomenon in natural populations of *E. tricuspidata* and lost legs can be regenerated; (2) autotomy reduces predation efficiency and impairs mating attempts and duration.

## 2. Materials and Methods

### 2.1. Insects

During April and November 2024, we conducted a general survey of *E. tricuspidata* (*N* = 894; female: *N* = 610; male: *N* = 284) in the shrubbery at Shahu Park, Wuhan City, Hubei Province, China (30.56° N, 114.34° E). The collection method was as follows: a sampling net was held beneath host plants, and a wooden stick was tapped on the leaf to guide spiders into the net. The number of spiders in each capture net was recorded. Each spider was placed in transparent enclosures (height × diameter: 6 cm × 2 cm) and kept under the same environmental conditions (28 ± 0.5 °C with 70% ± 10% relative humidity). Those spiders were fed fruit flies (*Drosophila melanogaster*) every 2 days.

### 2.2. Autotomy Treatment

Male and female spiders with intact legs were assigned to either the autotomy group or the control group, producing four combinations of experimental groups: (1) right second leg autotomy; (2) left second leg autotomy; (3) bilateral second leg autotomy; (4) control (no autotomy). Some individuals within the autotomy treatment were induced to autotomize a second pair of legs by gently grasping the leg at the femur with a pair of forceps. Control spiders were handled similarly but were not grasped with forceps. The second pair of appendages was chosen for manual autotomy because this species is more prone to losing that pair in natural populations.

### 2.3. Regeneration Trials

This experiment used third-instar juveniles with intact legs, with 20 individuals in each treatment group. Body size was measured before the experiment, and no significant differences were observed among groups. Artificial induction of autotomy was performed 24 h before the start of trials. Juveniles were selected for the regeneration trials because they have the ability to molt, which is essential for leg regeneration.

### 2.4. Predation Trials

Spiders were fasted for 48 h prior to the experiment to maximize feeding activity during the experiment [18]. Artificial induction of autotomy was performed 48 h before the start of predation trials. Before the experiment, the spiders were transferred individually to transparent test tubes, each containing 30 fruit flies. Four groups, each consisting of 20 adult spiders of similar body size, were established, and the number of fruit flies captured was recorded. They were recorded at 5 min, 10 min, 20 min, 30 min intervals.

### 2.5. Mating Trials

Because the male *E. tricuspidata* are the active courtship side, males that had self-amputated their appendage were selected for mating experiments. Each group consisted of 15 males and 15 females. Before testing, we would choose female spiders of the same size and age, which are of the same sexual maturity and age as male spiders. The time interval between artificial autotomy and mating trial is 48 h. One female was placed in a new opaque plexiglass mating arena (height × length × width: 7.5 cm × 5 cm × 5 cm) for a 30 min acclimation period, then one male was introduced. And the mating experiment was carried out at a fixed time every day. Spiders were continuously observed for 90 min, and we recorded instances of mating. If the male spider did not mate with the female within 30 min, the mating was defined as a failure. If more than 30 min have passed since one male mated with a female spider, the mating was considered to be over.

### 2.6. Data Analysis

Similar to previous papers [19,20,21,22], we first used the Shapiro–Wilk and Levene tests to verify the normality and variance homogeneity of the data. Chi-square tests (χ^2^) were used to test for differences in leg autotomy rates across months, sex, age, and so on. Predation efficiency was analyzed using a linear mixed model (LMM), including group, time, and the group × time interaction as fixed effects and individual ID as a random effect. One-way ANOVA was used to compare prey capture rates among groups at each time point separately. Welch’s one-way ANOVA was used to compare the differences in mating attempts and mating duration among each group. Regeneration rate, survival rate, and mating success rate were also used in chi-square tests (χ^2^). Post hoc pairwise comparisons for all significant multi-group tests were conducted using appropriate methods. All statistical analyses were performed using R 4.2.1 (R Core Team, 2023), and data were graphed using GraphPad Prism 10 (GraphPad Software, Boston, MA, USA). Data are presented as mean ± standard error (SE) unless otherwise stated.

## 3. Results

### 3.1. Incidence of Leg Autotomy in Natural Populations

**Figure 1:**
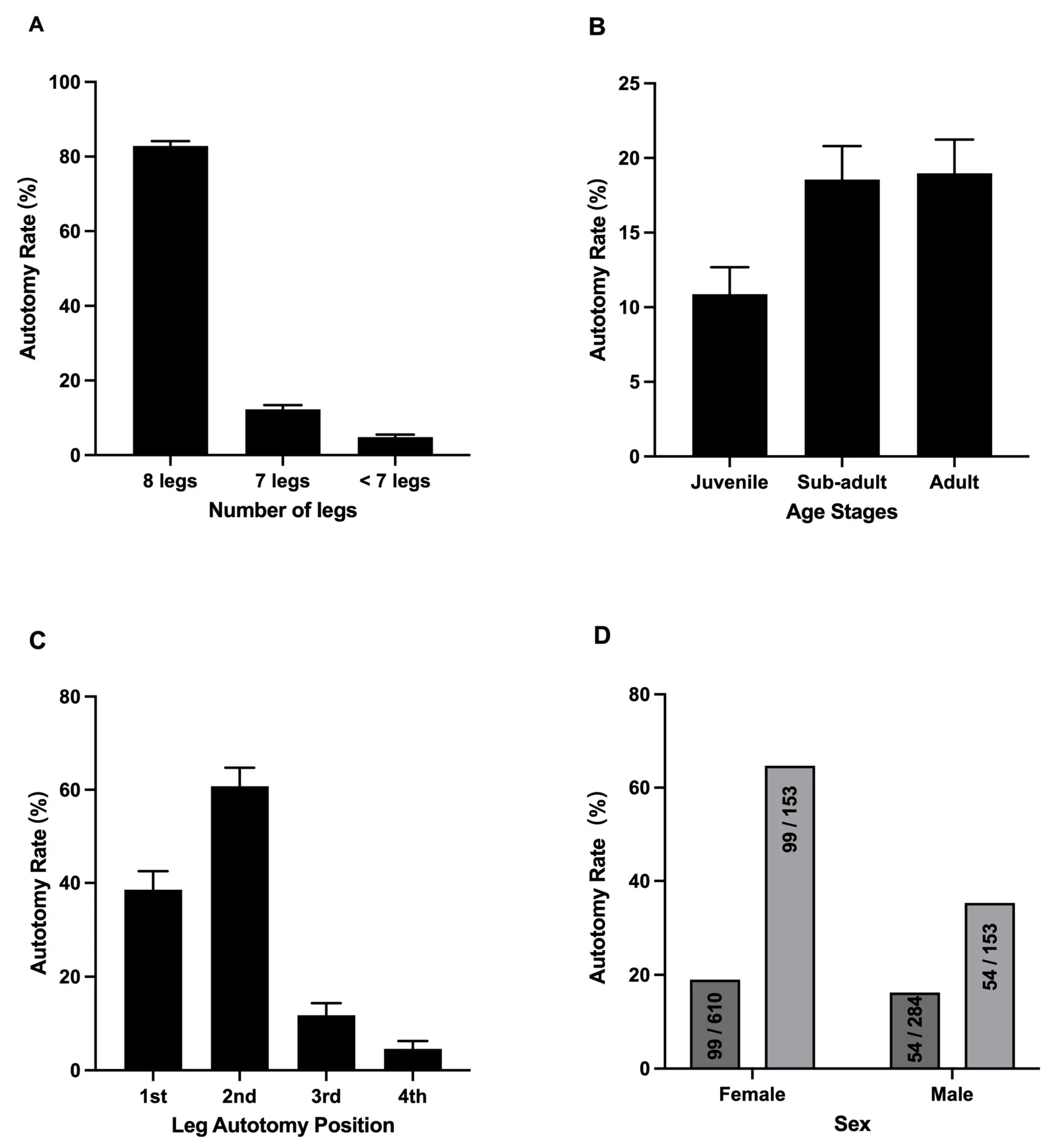
Leg autotomy in natural populations. (**A**) The number of leg autotomies in individuals that lose their legs (*N* = 894, χ^2^ = 25.6, df = 2, *p* < 0.001). (**B**) The probability of leg autotomy at different life stages (*N*: Juvenile = 193, Sub-adult = 248, Adult = 453; χ^2^ = 6.763, df = 2, *p* = 0.0340). (**C**) The location of autotomy in individuals that lose legs; if a single spider loses multiple legs, each lost leg counts as one individual (*N* = 153, χ^2^ = 35.6, df = 3, *p* < 0.001). (**D**) The probability of leg autotomy by sex; dark gray represents the rate of autotomized individuals to the total number of individuals of that sex, and light gray represents the rate of autotomized individuals to the total number of autotomized individuals (χ^2^ = 1.059, df = 1, *p =* 0.3034).

Based on the field surveys of all samples, 17.11% of *E. tricuspidata* are missing at least one leg. Within the natural population, there was a highly significant difference between individuals missing a single leg and those missing multiple legs (Figure 1A). Among all collected individuals, the autotomy rates for females and males accounted for 16.22% and 19.01% of their respective total populations, respectively, which did not differ significantly (Figure 1D). Among spiders missing legs, the probability of loss was similar for the left (49.67%) and right legs (51.63%). If a single spider loses multiple legs, count each lost leg once. Of spiders missing legs, the missing legs were more likely to occur in the first two pairs rather than the last two pairs, with the percentage of missing the second pair leg is 60.78% (Figure 1C). Adult individuals were more likely to be missing legs than juveniles, accounting for 18.98% (Figure 1B). Interestingly, there was a highly significant difference in the probability of autotomy among individuals across months, with a peak in July (χ^2^ = 23.6, df = 7, *p* < 0.001; Table 1).

### 3.2. Leg Regeneration Performances

**Figure 2:**
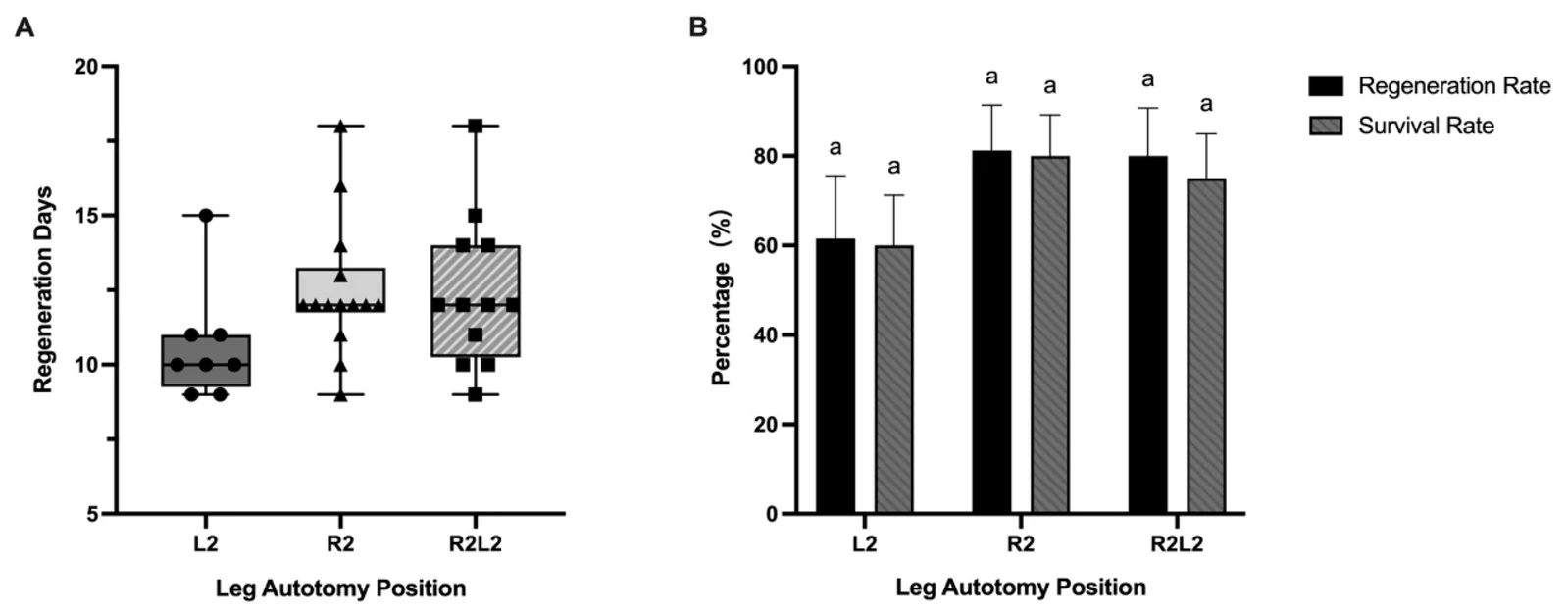
Regeneration behaviors in *E. tricuspidata*. (**A**) Differences in regeneration days at different positions following autotomy in the individuals’ development. Boxes denote the interquartile range, solid horizontal lines inside boxes represent median values, and whiskers extend to the maximum and minimum values within 1.5 × IQR. Each data point on the box plot represents the number of regeneration days for each individual. (**B**) Differences in regeneration rate and survival rates at different positions following autotomy in individuals. L2, left 2nd leg autotomy; R2, right 2nd leg autotomy; R2L2, bilateral 2nd legs autotomy. To distinguish mortality caused by autotomy from background mortality in the laboratory, we established a complete control group (CK) and found that the survival rate of intact spiders was 100% for 18 days. The differences in lower case letters above each bar indicate significant differences (*p* < 0.05).

**Figure 3:**
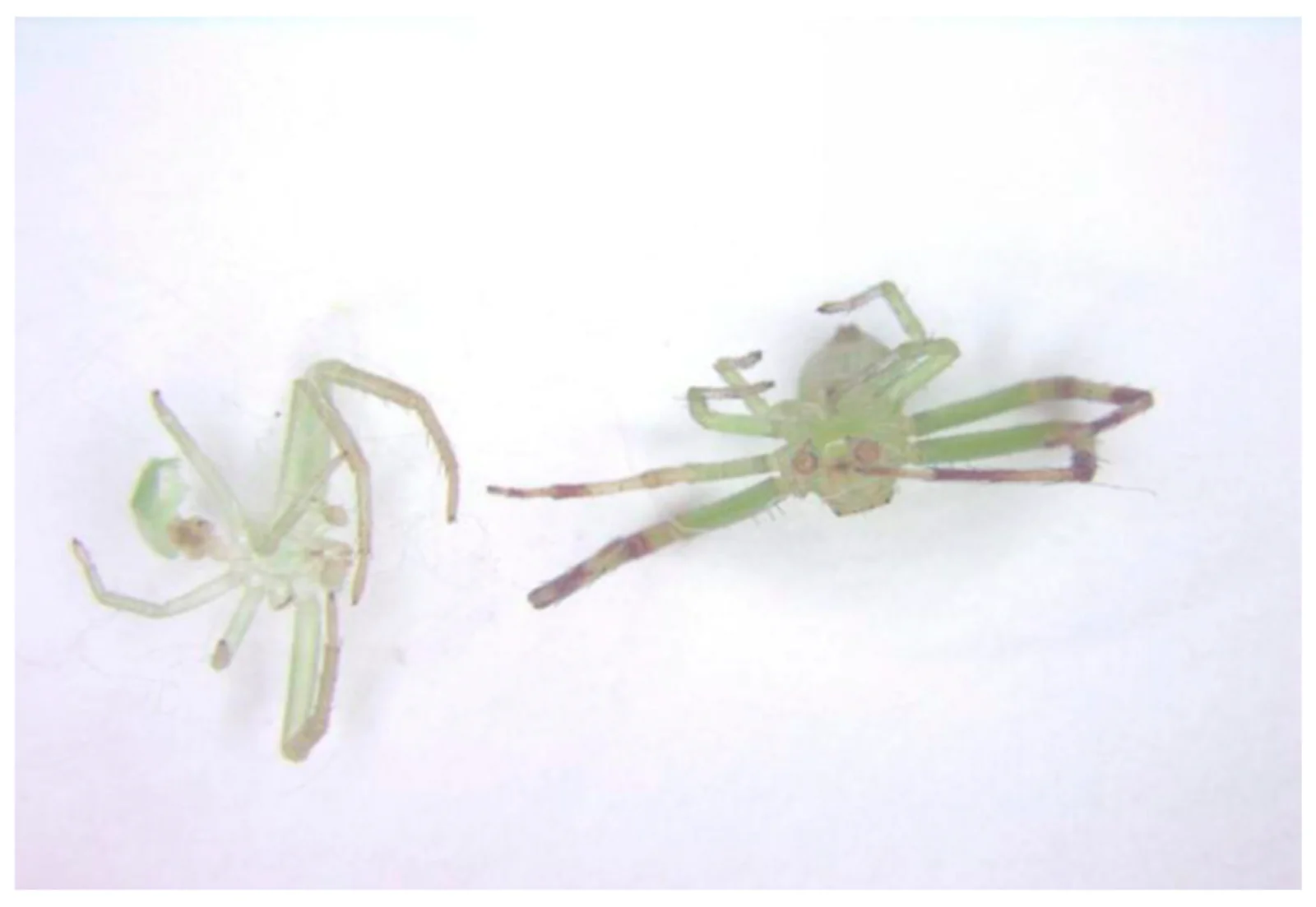
Molting and intact regenerated individuals of *E. tricuspidata*; on the left is the exuviae molted by an individual that lost the right appendage of its second pair, and on the right is the intact individual after molting and regeneration. An individual is a third-instar juvenile, and it took 10 days to regrow its leg.

In our experiment, following autotomy of the appendage in *E. tricuspidata*, appendage regeneration generally took 9–18 days, with no significant differences in regeneration duration observed between the different groups (W_2.000,18.93_ = 2.413, *p* = 0.1165; Figure 2A). As for the regeneration rate, it ranged from approximately 60% to 80%, with no significant differences (χ^2^ = 2.717, df = 4, *p* = 0.6063). The mortality rate among autotomized individuals ranged from 15% to 40% across treatments, with no significant difference in mortality among the treatment groups (χ^2^ = 2.134, df = 2, *p* = 0.3440, Figure 2B). All individuals capable of regenerating appendages underwent one molt (Figure 3).

### 3.3. The Effect of Autotomy on Predation Ability

**Figure 4:**
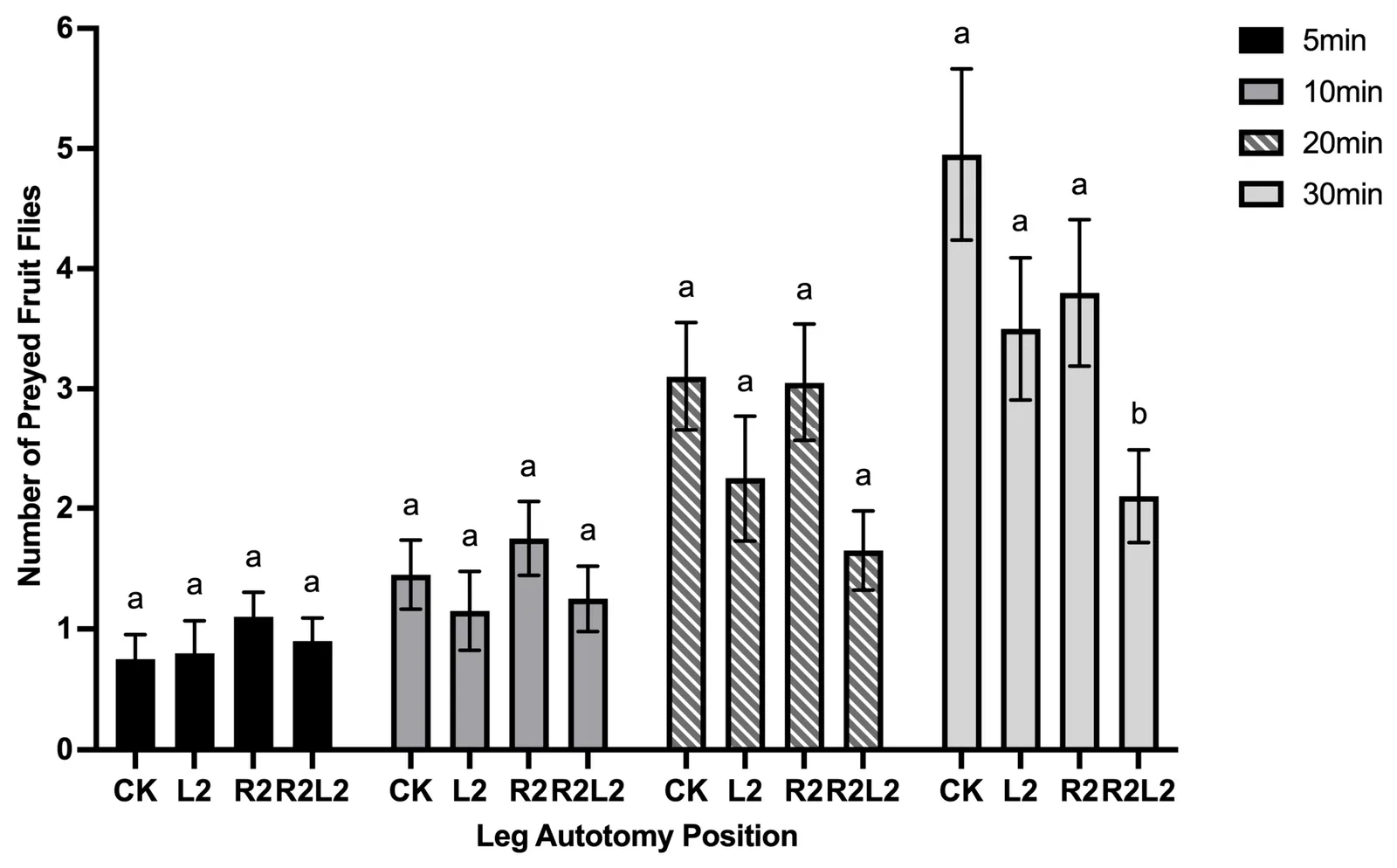
Effect of different leg autotomy positions on predation of *E. tricuspidata*; CK, control group; L2, left 2nd leg autotomy; R2, right 2nd leg autotomy; R2L2: bilateral 2nd leg autotomy. The differences in lower case letters above each bar indicate significant differences (*p* < 0.05).

All *E. tricuspidata* in each treatment group could prey on fruit flies within 5, 10, 20, and 30 min. Furthermore, within the first 5, 10, and 20 min, there was no significant difference in capture number between individuals missing one leg and those missing two legs. However, in 30 min, if *E. tricuspidata* missing a pair of legs would show significantly lower predation efficiency compared to intact individuals and those missing a single leg (*F*_(3,76)_ = 3.994, *p* = 0.0107, *R*^2^ = 0.1362; Figure 4). Meanwhile, according to LMM, time had a significant main effect on predation (*p* < 0.001), while the main effect of treatment group was not statistically significant (*p* = 0.9937), and the group × time interaction was also not significant (*p* = 0.9938).

### 3.4. The Effect of Autotomy on Mating Performances

**Figure 5:**
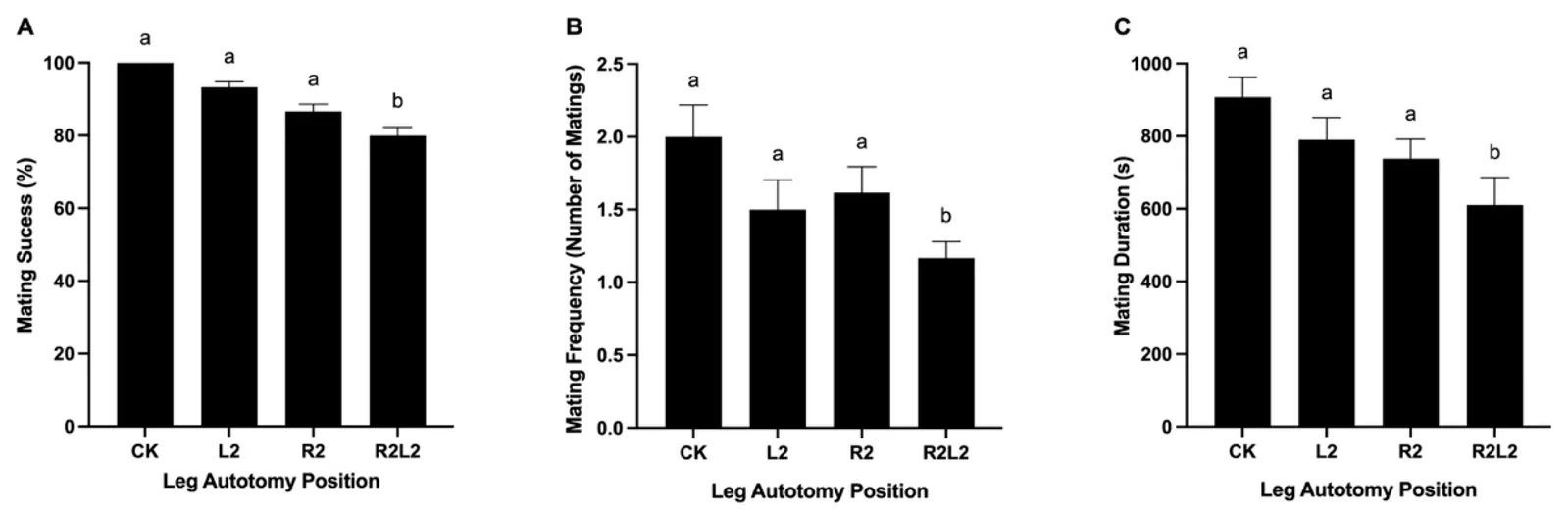
Effects of different leg autotomy positions on mating performance of *E. tricuspidata*. (**A**) Differences in mating success rates among leg autotomy positions. (**B**) Differences in the numbers of matings among leg autotomy positions. (**C**) Differences in mating duration among leg autotomy positions. CK, control group; L2, left 2nd leg autotomy; R2, right 2nd leg autotomy; R2L2, bilateral 2nd leg autotomy. The differences in lower case letters above each bar indicate significant differences (*p* < 0.05).

We conducted some experiments to compare mating performances between intact individuals and those with artificially amputated legs. It was found that intact individuals had a 100% mating success rate, whereas those missing a single leg had mating success rates decreased, showing no significant difference from intact individuals. For individuals missing a pair of legs, the mating success rate significantly decreased (χ^2^ = 16.08, df = 3, *p* = 0.0011; Figure 5A). *E. tricuspidata* with only one leg loss mated fewer frequency than intact, though no difference existed between these groups. However, individuals missing two legs exhibited extremely significantly fewer mating attempts than intact specimens (W_3.000,27.24_ = 4.282, *p* = 0.0134; Figure 5B). Regarding mating duration, there was a highly significant difference between intact individuals and those with higher leg loss, with deficient appendage individuals having markedly shorter mating times than intact individuals (W_3.000,27.08_ = 3.532, *p* = 0.0280; Figure 5C).

## 4. Discussion

Although leg autotomy is a typical anti-predation strategy in spiders, enabling them to increase their chances of escape when faced with predation threats [23], it may entail some fitness costs, and there is significant variation in the pattern of autotomy among individuals across different species and habitats. According to relevant research, crab spiders missing a foreleg are weaker than intact individuals in struggles for mates [9]. For *Agelenopsis aperta*, the loss of appendages due to web-defending conflicts affects the individual’s subsequent survival [24]. Some arthropods are capable of regeneration, but the mechanisms vary significantly among species, and regeneration may hinder growth and development [6]. In certain spiders, missing legs lost during the first quarter of the interval between molts may regenerate [13]. Overall, for individuals that invest resources in regeneration, leg autotomy carries a high cost. However, when faced with the choice between leg loss and death, leg autotomy appears to be the optimal choice.

### 4.1. Natural Populations and Responses to Environmental Stress

The rate of leg autotomy in wild populations of *E. tricuspidata* was approximately 17.11%, which falls within the typical range of autotomy frequency observed in spider populations [8] and is lower than that reported for *Pardosa valens* [25] and *Misumena vatia* [26], but is similar to that of most reported populations [27,28]. As an ambush spider, this moderate autotomy rate falls within the range reported for other crab spider species [29]. Our study found no difference in autotomy rate between the sexes, which contrasts with the higher incidence of autotomy typically observed in most hunting male spiders [30] and the presence of sexual cannibalism [31]. Unlike *Pholcus beijingensis* [32], *E. tricuspidata* is more prone to losing the second pair of legs at a higher rate, consistent with the higher exposure risk associated with its forelegs, which serve multiple functions, including predation, sensory detection, and courtship. Hunting spiders typically exhibit higher rates of leg autotomy than web-building spiders, a phenomenon that may be attributed to their reliance on locomotion and the functional roles of their front legs. Additionally, the autotomy rate shows significant seasonal variation, peaking from May to July, which may correlate with multiple co-occurring factors. Higher temperatures and increased activity levels during summer may elevate encounter rates with both prey and predators. When combined with habitat disturbances caused by humans, this may increase the risk of autotomy. A Study on *Neoscona vigilans* has shown that human disturbances significantly affect the species’ survival and development [33], but the mechanisms underlying autotomy rates in non-orb-webbing spiders differ from those of orb-webbing spiders and may involve direct physical injury and pesticide use. Autotomy rates in adult spiders are higher than in juveniles, which is consistent not only with the fact that adults cannot molt and regenerate but may also be related to the intensity of adult dispersal, courtship, and predation activities. This pattern is also observed in tropical wetland spiders [34]. Temperature and cold tolerance may also influence seasonal autotomy patterns [35], but the specific effects on the frequency and mechanism of autotomy in *E. tricuspidata* remain to be studied.

### 4.2. Regeneration Capacity and Functional Recovery from Injury

Leg regeneration of *E. tricuspidata* depends on the molting cycle, and the rate of regeneration is related to factors such as age, nutrition, and temperature [36]. Environmental factors such as temperature have a significant impact on regeneration success rates; the urban heat island can disrupt molting and regeneration in *Latrodectus hesperus* [37]. Most spiders require 1–2 molts to complete regeneration: In *Araneus diadematus*, regenerated appendages grow back after molting and quickly regain function [38], and *Davus pentaloris* can recover its running speed fully following regeneration [39]. In a study of *Pardosa pseudoannulata*, individuals were found to prioritize maintaining basic survival and foraging functions and to reduce their investment in reproduction [40]. Different species exhibit distinct regeneration strategies: *Pholcus beijingensis* does not regenerate appendages to conserve energy [32], and *Zygiella x-notata* also has the inability to regenerate, which may reflect a high tolerance for leg loss [27]. In contrast, *Nymphon brevirostre*, which belongs to the same subphylum as spiders, possesses exceptional regenerative capacity [41]. Our study found that some *E. tricuspidata* individuals regenerate lost legs after a single molt, thereby moderating regenerative recovery and energy expenditure, although the specific energy trade-offs require further research.

### 4.3. Temporal Dynamics of the Effects of Autotomy on the Predatory Efficiency

Spiders with different foraging strategies exhibit distinct antipredator responses. Web-hunting spiders of the *Trichonephila* rely on their web to ambush prey and use an extremely prolonged thanatosis to avoid attack [42]. In contrast, non-orb-webbing spiders such as *E. tricuspidata* may lose legs more frequently when encountering predators. In our study, there were no significant differences within a 30 min observation window, suggesting that rapid compensation can be achieved through behavioral adjustments. As reported for other spider species, *Schizocosa ocreata* can adjust the coordination of its remaining legs to maintain hunting efficiency [43], while *Pholcus manueli* can rapidly adjust its support angle and step frequency following autotomy [44]. However, when the duration exceeds 20 min, leg loss may increase energy expenditure and reduce stability, thereby impairing sustained hunting efficiency, similar compensatory adjustments have also been observed in *Myrmarachne gisti* [45]. It should be noted that our trials used fruit flies as prey. As omnivorous predators, spiders typically maintain consistent capture efficiency for small and active prey [46], and the impact of autotomy on predatory efficiency does not differ substantially across different prey types [25]. However, fruit flies have limited mobility, which may have mitigated the negative impact of autotomy on mobility. In their other natural habitats, more active prey may impose higher locomotor demands and reveal stronger effects of autotomy on predation efficiency.

### 4.4. Threshold Effects of Autotomy on the Mating and Reproductive Characteristics

In terms of reproductive and mating capacity, leg autotomy in *E. tricuspidata* exhibits a clear threshold effect: the loss of a single leg has a minimal impact, while the loss of multiple legs significantly reduces mating duration and frequency. A similar pattern of autotomy cost has also been observed in *Schizocosa ocreata* [47]. In male spiders, the forelegs not only serve a locomotor function but are also key organs for visual and vibrational communication. Leg loss may directly reduce the quality of courtship signals and female receptivity [48]. Recent work on *Schizocosa* wolf spiders has further demonstrated that both courtship displays and the signaling environment influence mating outcomes [49]. We hypothesize that the absence of legs may limit the flexibility of these behaviors. Our study found that the loss of two legs significantly shortens mating duration, which is consistent with findings in wolf spiders [50]. Some behavioral experiments on captive *Pardosa saltans* have shown that prolonged activity intensifies behavioral inhibition induced by structural defects [51]. Based on our study, we speculate that the threshold effect may be related to energy allocation between single- and multiple-leg losses. Some studies have suggested that wolf spiders face an energy allocation trade-off between regeneration and reproduction [39]. However, because our study did not directly measure sperm transfer, fertilization success rates, or energy expenditure, the specific mechanisms underlying the threshold effect in *E. tricuspidata* remain to be elucidated.

## 5. Conclusions

In summary, leg autotomy in *E. tricuspidata* represents an evolutionary trade-off between short-term anti-predation benefits and long-term functional costs, and the pattern of autotomy results from joint regulation by season and habitat. In the future, movement ecology could be employed to analyze the impact of autotomy on individual mobility, and studies on the fitness of offspring could be conducted to more comprehensively reveal the ecological significance of leg autotomy in crab spiders.

## Figures and Tables

**Table 1:** Monthly variation in leg autotomy rate in *E. tricuspidata*. Autotomy individuals represent the number of spiders collected that month with missing legs, total individuals represent the number of spiders collected that month, and autotomy rate represents the percentage of leg autotomy observed in spiders collected that month. Data are presented as mean ± standard error (SE).

Month	Autotomous Individuals (*N*)	Total Individuals (*N*)	Autotomy Rate (%)
April	4	30	13.33 ± 1.92
May	12	56	21.43 ± 2.35
June	26	104	25.00 ± 2.17
July	28	111	25.22 ± 2.16
August	21	126	15.67 ± 2.04
September	23	220	10.44 ± 1.49
October	35	207	16.91 ± 2.02
November	4	40	10.00 ± 1.94

## Data Availability

The data presented in this study are available upon request from the corresponding author.
